# Predicting treatment outcomes of intravitreal brolucizumab for polypoidal choroidal vasculopathy through noninvasive assessment of polypoidal lesion blood flow with optical coherence tomography angiography

**DOI:** 10.1038/s41598-024-51628-0

**Published:** 2024-01-10

**Authors:** Junki Hoshino, Hidetaka Matsumoto, Kosuke Nakamura, Hideo Akiyama

**Affiliations:** Department of Ophthalmology, Gunma University Graduate School of Medicine, 3-39-15 Showa-machi, Maebashi, Gunma 371-8511 Japan

**Keywords:** Diseases, Eye diseases, Macular degeneration

## Abstract

We investigated the assessment of blood flow within polypoidal lesions using optical coherence tomography angiography (OCTA) to determine intravitreal brolucizumab (IVBr) efficacy for treating polypoidal choroidal vasculopathy (PCV). We retrospectively studied 46 eyes with PCV that completed 1-year IVBr treatment. Blood flow signals within polypoidal lesions were evaluated using OCTA after loading-phase treatment, and 1-year outcomes were compared between eyes in which blood flow signals disappeared versus persisting. After loading-phase treatment, blood flow signals within polypoidal lesions disappeared in 31 eyes and persisted in 15. In the former group, visual acuity improved significantly throughout the year (*P* < 0.01), while in the latter there was no significant difference between baseline and after 1 year. The total number of injections was significantly lower with than without disappearance of blood flow signals (6.0 vs. 6.9, *P* < 0.01). The intended injection interval at the last visit was significantly longer in the former than in the latter group (15.7 weeks vs. 12.5 weeks, *P* < 0.01). These results indicate that PCV cases showing disappearance of blood flow signals within polypoidal lesions by OCTA after loading-phase treatment had favorable 1-year outcomes of IVBr. Therefore, evaluating blood flow within polypoidal lesions by OCTA may allow noninvasive prediction of PCV treatment outcomes.

## Introduction

Polypoidal choroidal vasculopathy (PCV), first described by Yannuzzi et al.^1^ as idiopathic polypoidal choroidal vasculopathy, has traditionally been classified as a subtype of neovascular age-related macular degeneration (nAMD). Recently, however, the nAMD nomenclature study group has endeavored to redefine and standardize the terms associated with nAMD^2^. According to their report, PCV can be classified as a subtype of type 1 macular neovascularization (MNV), with branching vascular networks (BVN) and aneurysmal dilations. Previous studies have shown PCV to be more common in Asians than in Caucasians^3–5^. Moreover, pachychoroid has been suggested to be associated with the development of PCV^6^, which has recently attracted major research attention.

The most characteristic finding of PCV is the presence of polypoidal lesions. Polypoidal lesions are observed as orange-red lesions on ophthalmoscopy. However, indocyanine green angiography (ICGA) is regarded as the gold standard modality for diagnosing PCV because it shows the higher sensitivity for detecting polypoidal lesions^7^. However, performing ICGA frequently is not practical because it is an invasive modality with the possibility of allergic reactions and requires a relatively long examination time. In addition, the availability of ICGA may be limited in some clinical practices. Therefore, there has been a trend toward diagnosing PCV without ICGA. The Asia–Pacific Ocular Imaging Society PCV Workgroup reported that a combination of three major optical coherence tomography (OCT)-based criteria, i.e., sub–retinal pigment epithelium (RPE) ring-like lesion, en face OCT complex RPE elevation, and sharp-peaked RPE detachment (PED), achieved 82% accuracy for the diagnosis of PCV^8^. In recent years, the advent of optical coherence tomography angiography (OCTA) has enabled the performance of noninvasive, high-definition, three-dimensional evaluation of retinochoroidal blood flow, further increasing the momentum for non-ICGA-based PCV diagnosis. In the early period of OCTA application, it was not feasible to fully replace ICGA when diagnosing PCV because the detection rate of polypoidal lesions on the en face OCTA images varied among reports^9–16^. Recently, however, OCTA B-scan images were reported to improve the detection rate of polypoidal lesions and are now expected to facilitate the diagnosis of PCV^17,18^.

Polypoidal lesions can cause severe vision loss due to the development of extensive submacular hematoma^19^. Moreover, we have reported that the regression of polypoidal lesions reduces the number of anti-vascular endothelial growth factor (VEGF) injections needed and allows a longer injection interval for PCV treatment^20,21^. Thus, regression of polypoidal lesions is one of the goals of treating PCV. Assessing polypoidal lesions without ICGA has previously represented a significant clinical challenge. However, the advent of OCTA now allows blood flow within polypoidal lesions to be evaluated noninvasively. Therefore, we investigated the utility of assessing blood flow within polypoidal lesions using OCTA to determine the efficacy of PCV treatment. Herein, we investigated the blood flow within polypoidal lesions after loading-phase treatment with intravitreal brolucizumab (IVBr) for treatment-naïve PCV using OCTA B-scan images. We also evaluated the relationship between the presence of blood flow in polypoidal lesions and the 1-year treatment outcomes.

## Results

Forty-six eyes of 46 patients (35 eyes of 35 men and 11 eyes of 11 women, age: 73.3 ± 8.0 (74.5 (54, 87)) years) were included in this study. At baseline, blood flow signals within polypoidal lesions were detected by OCTA in all 46 eyes. After 3 months, blood flow signals within polypoidal lesions had disappeared in 31 eyes (67.4%, signal disappearance group) but were still present in 15 eyes (32.6%, signal persistence group). Baseline data, including age, gender, best-corrected visual acuity (BCVA), foveal thickness (FT), central choroidal thickness (CCT), and height of the polypoidal lesion, did not differ significantly between these two groups (Table 1).

**Table 1:** Baseline demographic and clinical findings of eyes with PCV that completed one year of intravitreal brolucizumab treatment.

	Signal disappearance group	Signal persistence group	*P* value
No. of eyes	31	15	
Age (years)	73.9 ± 7.4 (75 (56, 87))	72.0 ± 9.1 (74 (54, 85))	0.68
Male	23 (74.2%)	12 (80.0%)	0.48
BCVA (logMAR)	0.27 ± 0.30 (0.22 (− 0.08, 1.10))	0.16 ± 0.17 (0.15 (− 0.08, 0.40))	0.30
Foveal thickness (µm)	260 ± 106 (248 (113, 577))	274 ± 93 (273 (97, 435))	0.49
Central choroidal thickness (µm)	218 ± 90 (206 (77, 472))	302 ± 171 (249 (87, 752))	0.11
Height of polypoidal lesion (µm)	279 ± 149 (213 (93, 678))	331 ± 145 (314 (168, 725))	0.17

*PCV* Polypoidal choroidal vasculopathy; *BCVA* Best-corrected visual acuity.

Age, BCVA, foveal thickness, central choroidal thickness, height of polypoidal lesion: average ± standard deviation (median (min, max)).

**Figure 1:**
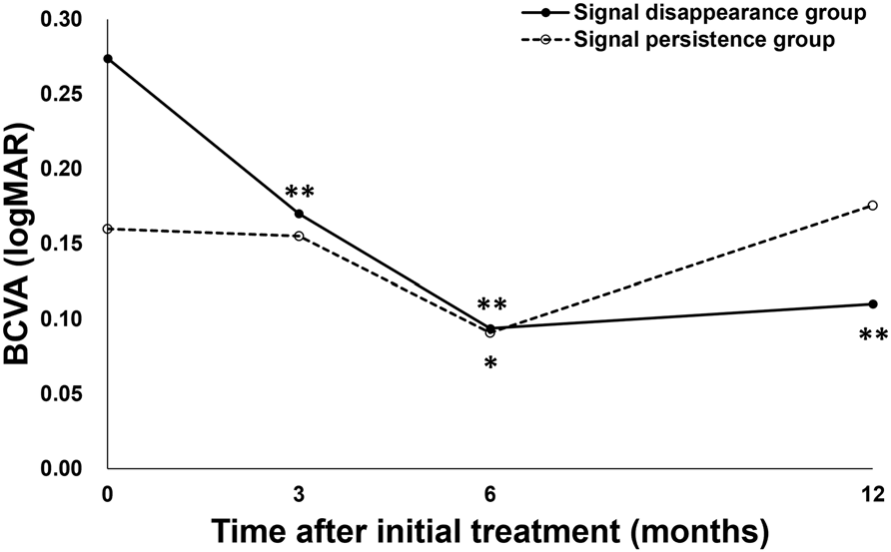
Changes of average best-corrected visual acuity in eyes with polypoidal choroidal vasculopathy treated with 3 monthly intravitreal injections of brolucizumab followed by a treat-and-extend regimen with intravitreal brolucizumab. Best-corrected visual acuity (BCVA) significantly improved throughout the 1-year study period in the group showing disappearance of blood flow signals, i.e., blood flow signals within polypoidal lesions detected by optical coherence tomography angiography disappeared after the loading phase treatment (**P < 0.01). On the other hand, in the group showing persistence of blood flow signals, in which blood flow signals within polypoidal lesions persisted after the loading phase treatment, BCVA showed significant improvement at 6 months after the initial treatment (*P < 0.05), while there was no difference between the values at baseline and after 1 year.

**Figure 2:**
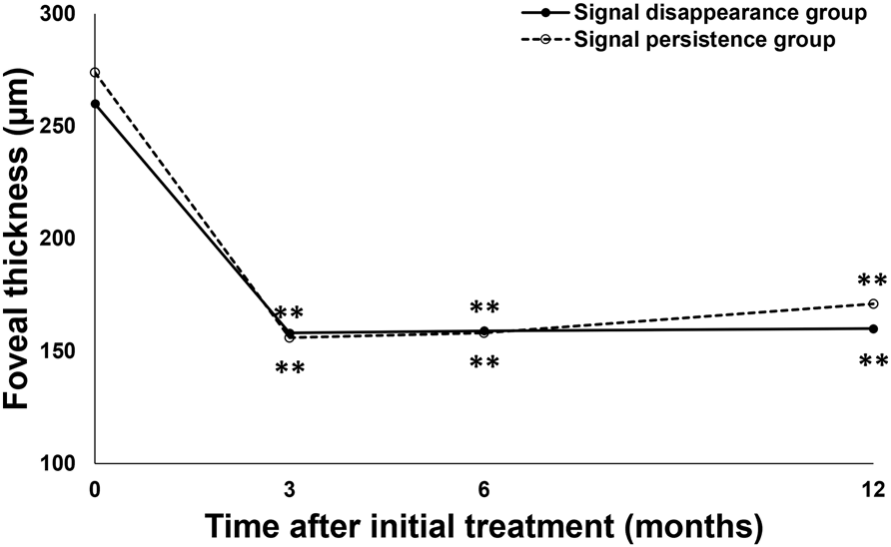
Changes in average foveal thickness in eyes with polypoidal choroidal vasculopathy treated with 3 monthly intravitreal injections of brolucizumab followed by a treat-and-extend regimen with intravitreal brolucizumab. Foveal thickness (FT) was significantly reduced and maintained during the 1-year study period in both the group showing disappearance of blood flow signals and that without such findings (**P < 0.01). The FT reductions did not differ significantly between the two groups.

**Figure 3:**
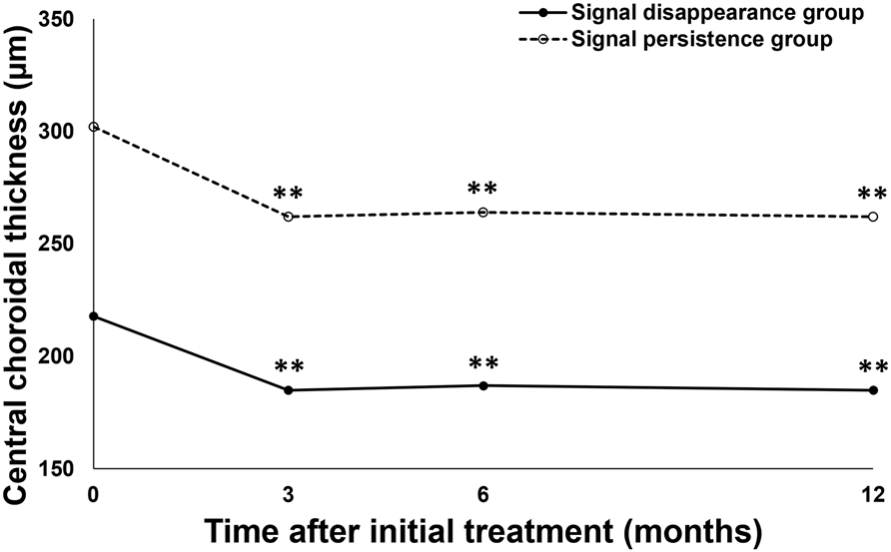
Changes in average central choroidal thickness in eyes with polypoidal choroidal vasculopathy treated with 3 monthly intravitreal injections of brolucizumab followed by a treat-and-extend regimen with intravitreal brolucizumab. Central choroidal thickness (CCT) was significantly reduced and the reduction was maintained during the 1-year study period in both the group showing disappearance of blood flow signals and that without such findings (**P < 0.01). The CCT reductions did not differ significantly between the two groups.

BCVA significantly improved from 0.27 ± 0.30 (0.22 (− 0.08, 1.10)) at baseline to 0.11 ± 0.29 (− 0.08 (− 0.08, 1.05)) units at 1 year in the signal disappearance group (*P* < 0.01), whereas the values were 0.16 ± 0.17 (0.15 (− 0.08, 0.40)) at baseline and 0.18 ± 0.33 (0.10 (− 0.08, 1.22)) at 1 year in the signal persistence group, showing no significant improvement (*P* = 0.56) (Fig. 1). BCVA at 1 year tended to be better in the signal disappearance group than in the signal persistence group (*P* = 0.07). FT at baseline and 1 year were 260 ± 106 (248 (113, 577)) μm and 160 ± 46 (160 (60, 249)) μm in the signal disappearance group and 274 ± 93 (273 (97, 435)) μm and 171 ± 51 (167 (96, 295)) μm in the signal persistence group, respectively, showing significant reductions in both groups (*P* < 0.01) (Fig. 2). CCT at baseline and 1 year were 218 ± 90 (206 (77, 472)) μm and 185 ± 81 (167 (53, 411)) μm in the signal disappearance group and 302 ± 171 (249 (87, 752)) μm and 262 ± 166 (210 (76, 691)) μm in the signal persistence group, respectively, showing significant reductions in both groups (*P* < 0.01) (Fig. 3). The reductions of FT and CCT did not differ significantly between the two groups (FT: *P* = 0.84, CCT: *P* = 0.17). Moreover, FT and CCT at 1 year were similar in the two groups (FT: *P* = 0.57, CCT: *P* = 0.19). The total number of injections during the 1-year study period was significantly lower in the signal disappearance group (*P* < 0.01), with a minimum of 6.0 in all cases in the signal disappearance group and 6.9 ± 0.9 (7 (6, 8)) in the signal persistence group. The intended injection interval at the last visit was 15.7 ± 1.0 (16 (12, 16)) weeks in the signal disappearance group and 12.5 ± 3.8 (16 (8, 16)) weeks in the signal persistence group, i.e., significantly longer in the former group (*P* < 0.01). The treatment outcomes after 1 year are summarized in Table 2.

**Table 2:** Summary of treatment outcomes after 1 year in signal disappearance and persistence groups.

	Signal disappearance group	Signal persistence group	*P* value
BCVA (logMAR)	0.11 ± 0.29 (− 0.08 (− 0.08, 1.05))	0.18 ± 0.33 (0.10 (− 0.08, 1.22))	0.07
Foveal thickness (µm)	160 ± 46 (160 (60, 249))	171 ± 51 (167 (96, 295))	0.57
Central choroidal thickness (µm)	185 ± 81 (167 (53, 411))	262 ± 166 (210 (76, 691))	0.19
Total number of injections	6.0 (in all cases)	6.9 ± 0.9 (7 (6, 8))	< 0.01
Intended injection interval at the last visit (week)	15.7 ± 1.0 (16 (12, 16))	12.5 ± 3.8 (16 (8, 16))	< 0.01

*BCVA* Best-corrected visual acuity.

Values: average ± standard deviation (median (min, max)).

**Figure 4:**
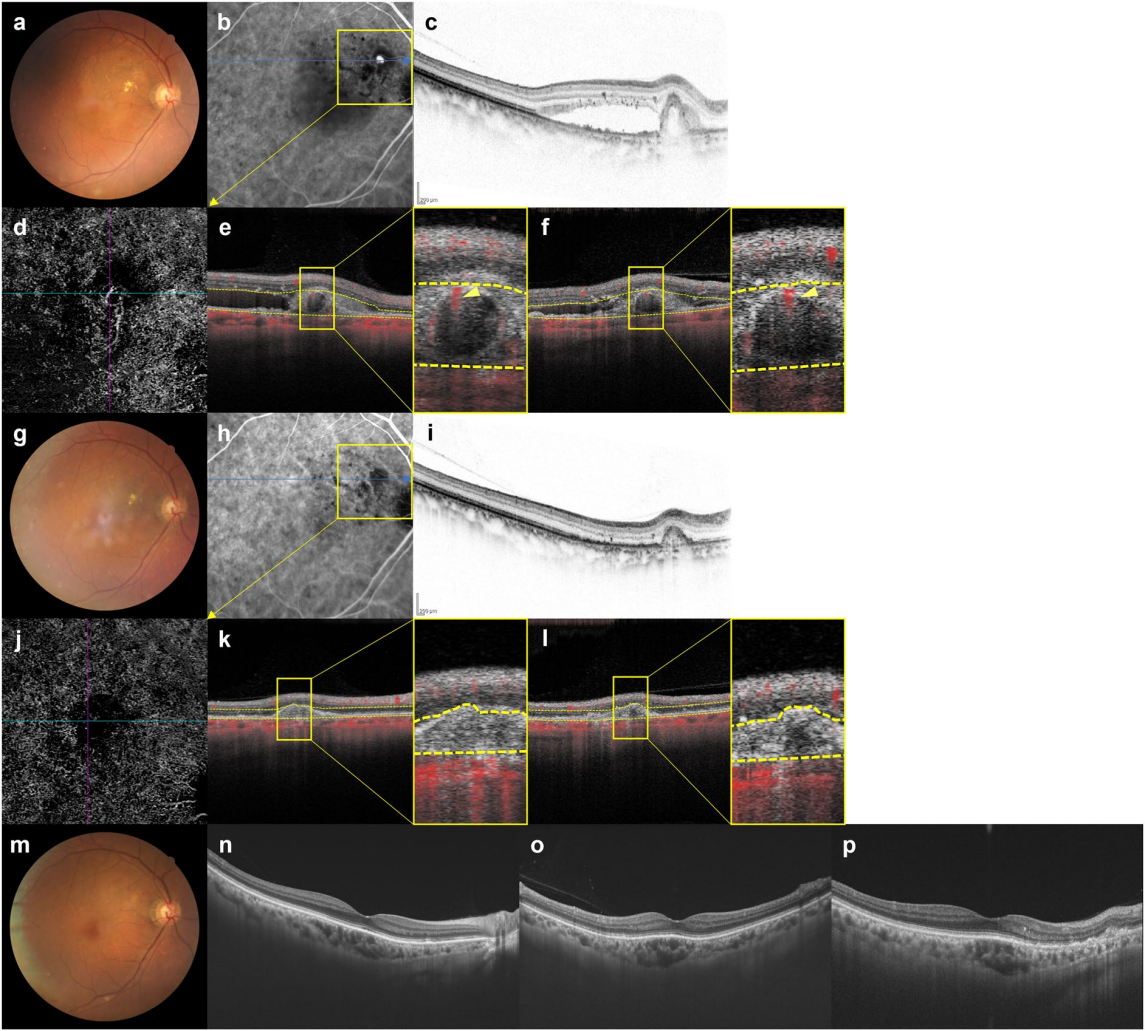
Images of the right eye of an 87-year-old man with polypoidal choroidal vasculopathy in the group with disappearance of blood flow signals within polypoidal lesions. (**a**–**f**): Baseline. Best corrected-visual acuity (BCVA) of the right eye is 0.16 logarithm of the minimal angle of resolution (logMAR) units. (**a**) Color fundus photograph shows a protruding lesion with serous retinal detachment (SRD) and hard exudates superotemporal to the optic disk. (**b**) Indocyanine green angiography (ICGA) shows a polypoidal lesion with a branching neovascular network (BVN). (**c**) Optical coherence tomography (OCT) B-scan image through the polypoidal lesion shows a sharp-peaked pigment epithelial detachment (PED) with SRD. (**d**) OCT angiography (OCTA) en face image shows blood flow signals corresponding to the polypoidal lesion and the BVN. (**e**, **f**) OCTA B-scan images (**e**: horizontal, **f**: vertical) show blood flow signal in the sub-RPE ring-like structure corresponding to the polypoidal lesion (arrowheads). (**g**–**l**) 3 months after the initial treatment. BCVA of the right eye is − 0.08 logMAR units. (**g**) Color fundus photograph shows no SRD. (**h**) ICGA shows complete regression of the polypoidal lesion. (**i**) OCT B-scan image shows the sharp-peaked PED to be diminished without SRD. (**j**) OCTA en face image shows no blood flow signal corresponding to the polypoidal lesion and the BVN. (**k**, **l**) OCTA B-scan images (**k**: horizontal, **l**: vertical) show no blood flow signal at the edge of the PED, which had been detected at baseline. (**m**–**p**) One year after the initial treatment. BCVA of the right eye is − 0.08 logMAR units. In total, 6 injections were given during the 1-year study period. (**m**) Color fundus photograph shows no exudative changes. (**n**–**p**) OCT B-scans through the fovea (**n**: horizontal, **o**: vertical, **p**: radial through the regressed polypoidal lesion) show dry macula.

**Figure 5:**
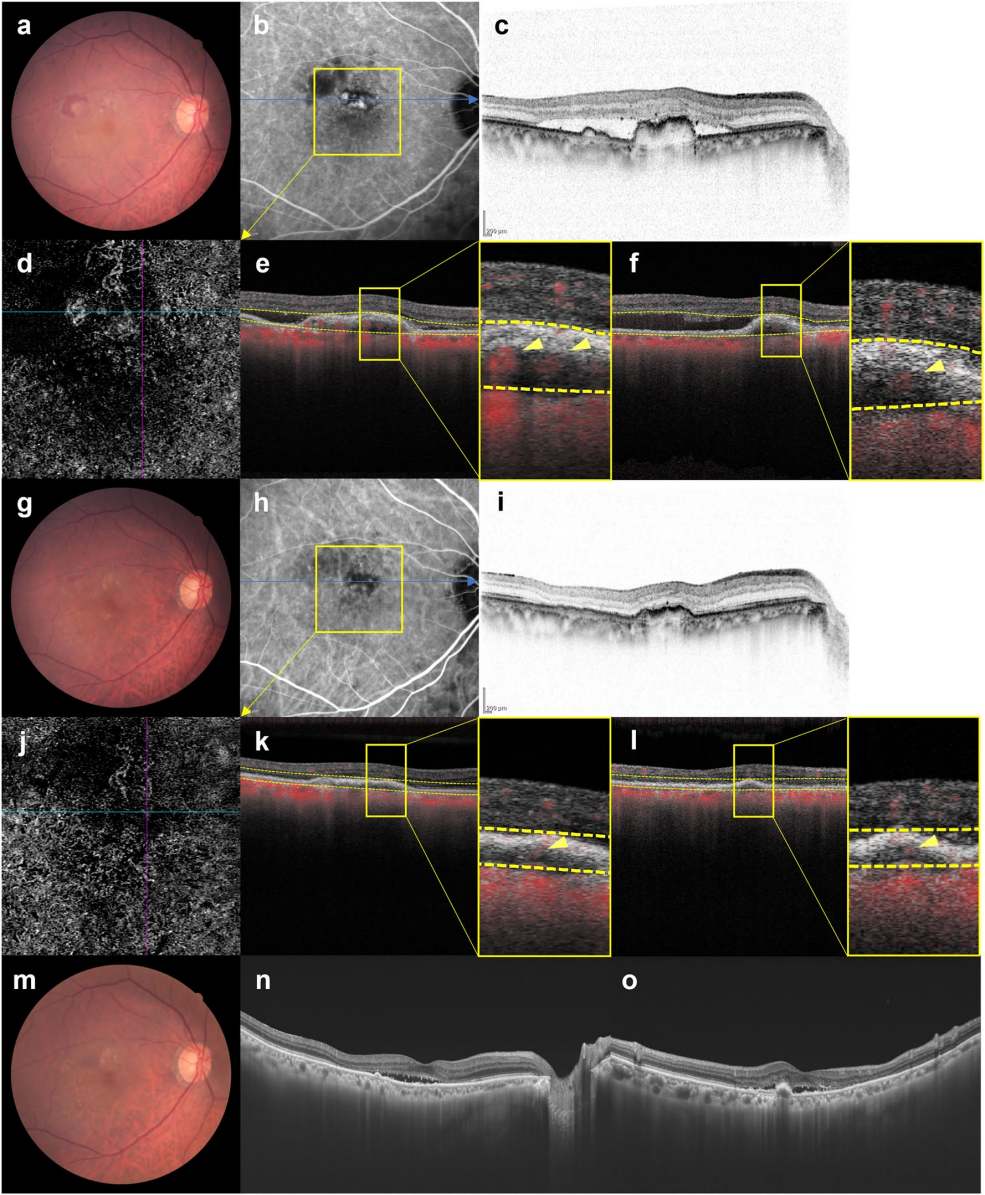
Images of the right eye of an 81-year-old man with polypoidal choroidal vasculopathy in the group showing persistence of blood flow signals within polypoidal lesions. (**a**–**f**) Baseline. Best corrected-visual acuity (BCVA) of the right eye is 0 logarithm of the minimal angle of resolution (logMAR) units. (**a**) Color fundus photograph shows a serous retinal detachment (SRD) and subretinal hemorrhage. (**b**) Indocyanine green angiography (ICGA) shows polypoidal lesions with a branching neovascular network (BVN) superior to the fovea. (**c**) Optical coherence tomography (OCT) B-scan image through the polypoidal lesions shows pigment epithelial detachments (PEDs) with SRD. (**d**) Optical coherence tomography angiography (OCTA) en face image shows blood flow signal corresponding to the polypoidal lesion and the BVN. (**e**, **f**) OCTA B-scan images (**e**: horizontal, **f**: vertical) show blood flow signals within the PED corresponding to the polypoidal lesions (arrowheads). (**g–l**) 3 months after the initial treatment. BCVA of the right eye is 0 logMAR units. (**g**) Color fundus photograph shows diminished subretinal hemorrhage without SRD. (**h**) ICGA shows persistent polypoidal lesions. (**i**) OCT B-scan image shows PED to be diminished without SRD. (**j**) OCTA en face image shows unclear blood flow signals within the polypoidal lesions. (**k**, **l**) OCTA B-scan images (**k**: horizontal, **l**: vertical) show blood flow signals within the PED corresponding to the polypoidal lesions (arrowheads). (**m–o**) One year after the initial treatment. BCVA of the right eye is 0 logMAR units. In total, 8 injections were required during the 1-year study period due to SRD recurrence. (**m**) Color fundus photograph shows no hemorrhage. (**n**, **o**) OCT B-scan images through the fovea (**n**: horizontal, **o**: vertical) show an SRD.

**Figure 6:**
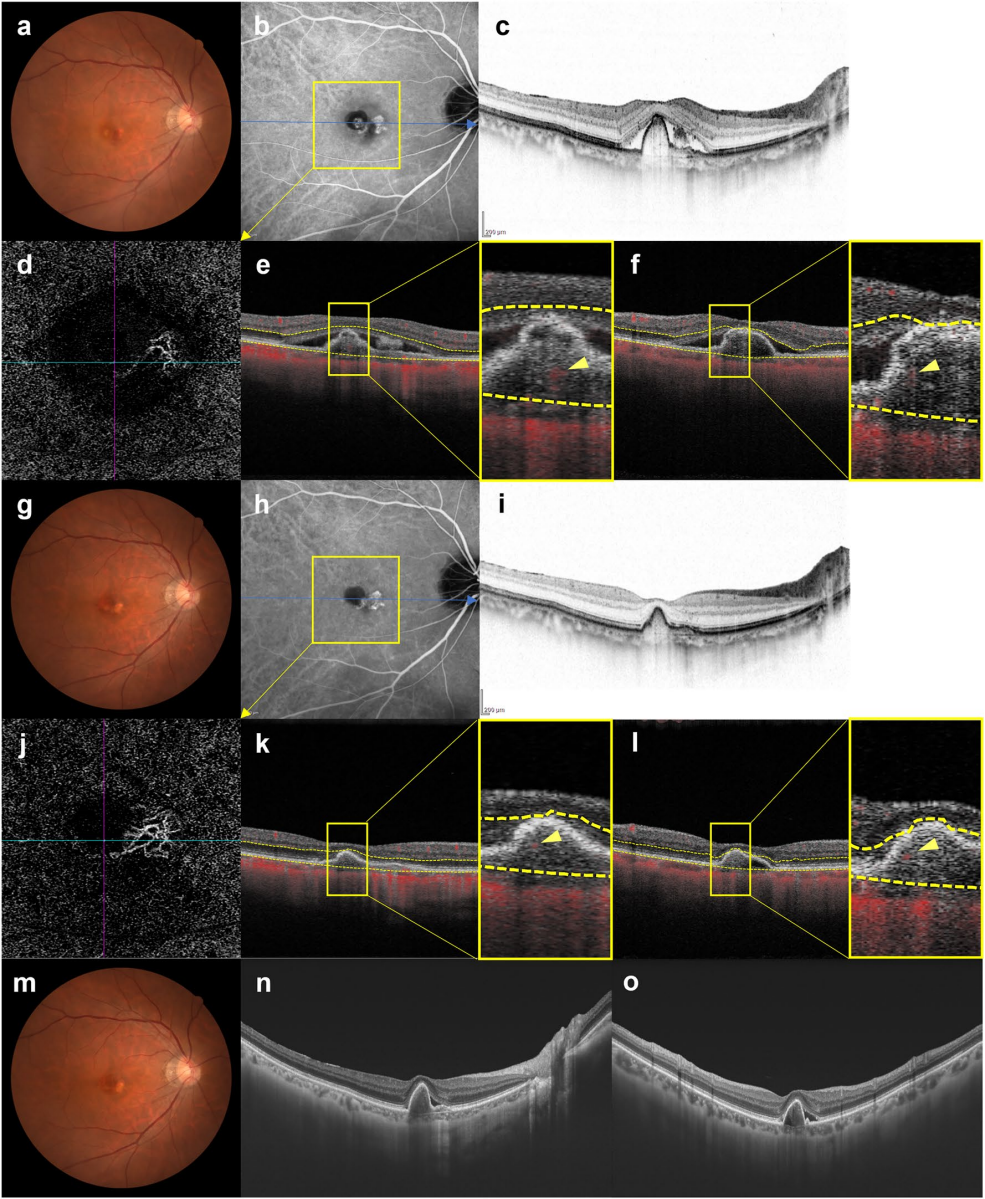
Images of the right eye of a 69-year-old man with polypoidal choroidal vasculopathy in the group showing persistence of blood flow signals within polypoidal lesions. (**a–f**) Baseline. Best corrected-visual acuity (BCVA) of the right eye is − 0.08 logarithm of the minimal angle of resolution (logMAR) units. (**a**) Color fundus photograph shows an orange-red lesion accompanied by subretinal hemorrhage. (**b**) Indocyanine green angiography (ICGA) shows a polypoidal lesion with a branching neovascular network (BVN). (**c**) Optical coherence tomography (OCT) B-scan image through the fovea shows a sharp-peaked and shallow irregular pigment epithelial detachment (PED) with serous retinal detachment (SRD). (**d**) Optical coherence tomography angiography (OCTA) en face image shows blood flow signals corresponding to the polypoidal lesion and the BVN. (**e**, **f**) OCTA B-scan images (**e**: horizontal, **f**: vertical) show blood flow signals within the PED corresponding to the polypoidal lesion (arrowheads). (**g**–**l**) 3 months after the initial treatment. BCVA of the right eye is − 0.08 logMAR units. (**g**) Color fundus photograph shows no subretinal hemorrhage. (**h**) ICGA shows complete regression of the polypoidal lesion. (**i**) OCT B-scan shows the sharp-peaked PED to be diminished without SRD. (**j**) OCTA en face image shows unclear blood flow signals within the polypoidal lesion. (**k**, **l**) OCTA B-scan images (**k**: horizontal, **l**: vertical) show blood flow signals within the PED corresponding to the polypoidal lesion (arrowheads). (**m–o**) 6 months after the initial treatment. BCVA of the right eye is − 0.08 logMAR units. (**m**) Color fundus photograph shows no subretinal hemorrhage. (**n**, **o**) OCT B-scan images through the fovea (**n**: horizontal, **o**: vertical) show SRD recurrence and an enlargement of the sharp-peaked PED. In total, 8 injections were required during the 1-year study period due to SRD recurrence.

After 3 months, ICGA showed that polypoidal lesions had completely regressed in 36 eyes (78.3%) while residual lesions were noted in 10 eyes (21.7%). Blood flow signals were detected by OCTA in all 10 eyes in which ICGA showed residual polypoidal lesions, and in 5 of 36 eyes (13.9%) in which polypoidal lesions had completely regressed. In the 5 eyes in which blood flow signals were detected by OCTA despite polypoidal lesions having completely regressed on ICGA after 3 months, BCVA at baseline and 1 year were 0.02 ± 0.11 (0.00 (− 0.08, 0.22)) and − 0.02 ± 0.12 (− 0.08 (− 0.08, 0.22)), FT were 201 ± 75 (208 (97, 318)) µm and 147 ± 29 (137 (121, 199)) µm, and CCT were 271 ± 168 (249 (87, 569)) µm and 240 ± 163 (210 (76, 531)) µm, respectively. The total number of injections during the 1-year study period was 6.6 ± 0.8 (6 (6, 8)), and the intended injection interval at the last visit was 16 weeks in all 5 of these eyes. Representative cases are shown in Figs. 4, 5 and 6.

## Discussion

We evaluated the blood flow signals within polypoidal lesions by OCTA after loading phase treatment with IVBr for PCV, and compared the 1-year treatment outcomes of IVBr between the blood flow signal disappearance and persistence groups. The group in which the flow signals disappeared showed significant visual improvement throughout the year, while there was no difference in VA between baseline and 1 year after the initial treatment in the group in which flow signals were still present. Moreover, the group showing disappearance of blood flow signals required significantly fewer injections during the 1-year study period and the intended injection intervals at the last visit were significantly longer than in the group with persistent blood flow signals.

Regression of polypoidal lesions is regarded as being one of the major goals of treating PCV. We previously investigated the correlation between the regression of polypoidal lesions and the outcomes of anti-VEGF treatment for PCV. We studied the 2-year outcomes of a treat-and-extend (TAE) regimen with intravitreal aflibercept (IVA) for PCV, and reported that cases in which the polypoidal lesions regressed after the loading-phase treatment required significantly fewer injections during the 2-year study period than those in which the lesions were residual^20^. In a study evaluating 1-year outcomes of a TAE regimen using IVBr for nAMD with type 1 MNV, we also found that PCV cases with regression of polypoidal lesions after the loading-phase treatment required significantly fewer injections during the treatment period and had significantly longer intended injection intervals at the last visit than cases with residual polypoidal lesions^21^. Several studies have evaluated the post-treatment blood flow signals within polypoidal lesions in PCV using OCTA B-scan images. Chang et al. evaluated the blood flow signals within polypoidal lesions employing spectral-domain OCTA (Avanti; Optovue, Fremont, CA, USA) after loading-phase treatment with 3 monthly IVA^22^. They reported that the cases in which the blood flow signals within polypoidal lesions were low or equivocal after the loading phase treatment had a significantly higher rate of subretinal fluid resolution than those showing high flow signals. Fukuyama et al. observed blood flow signals within polypoidal lesions by swept-source (SS) OCTA (DRI OCT-1 Triton) after administering a combination of IVA and photodynamic therapy (PDT) and described blood flow signals in polypoidal lesions at 2 weeks after this treatment as being significantly associated with recurrence or a poor response to the treatment^23^. In the current study, we treated PCV cases with brolucizumab which is a relatively new anti-VEGF agent, and evaluated the blood flow signals within polypoidal lesions employing the PLEX Elite 9000, an SS-OCTA, after the loading-phase treatment. Cases in which the blood flow signals within polypoidal lesions disappeared after the loading phase treatment required significantly fewer injections and had significantly longer intended injection intervals at the last visit than cases in which the blood flow signals persisted. BCVA did not differ significantly between the two groups either at baseline or at 1 year. However, significant BCVA improvement was maintained throughout the 1-year study period in the group showing disappearance of blood flow signals, but not in the group showing persistence of blood flow signals. This might be attributable to the baseline BCVA being poorer, relatively, in the signal disappearance group than in the signal persistence group as well as the lower recurrence frequency in the signal disappearance group. Thus, the blood flow signals within polypoidal lesions detected by OCTA might be associated with PCV treatment outcomes, such that OCTA may contribute to predicting the outcomes of noninvasively treating PCV.

The regression rates of polypoidal lesions evaluated by ICGA are reportedly about 30% for intravitreal ranibizumab (IVR)^24,25^, approximately 50% for IVA^20,26^, and 70–90% for combination therapy with PDT and IVR or IVA^24,25,27^, while approaching 80% for IVBr^21,28,29^. In previous studies that evaluated blood flow signals within polypoidal lesions using the OCTA B-scan modality, the rate of disappearance of blood flow signals within polypoidal lesions at 3 months after the initial treatment was 37.5% for IVA^22^ and 45.7% for combination therapy with PDT and IVA^23^, whereas the rate was 67.4% in the present study using IVBr. Therefore, brolucizumab appears to be effective for achieving regression of polypoidal lesions, leading to the favorable treatment outcomes for PCV.

Previous studies using OCTA en face images have shown relatively high detection rates of 70–100% for BVN, but the detection rates for polypoidal lesions were relatively low and highly variable, ranging from 17 to 85%^9–16^. Recently, Cheung et al. reported that the detection rate of polypoidal lesions can be improved from 43.5 to 82.6% by using structural OCT and OCTA B-scan images in addition to en face images^17^. Fujita et al. also reported that 94.7% of polypoidal lesions were detectable using the OCTA B-scan images^18^. The difficulty in evaluating polypoidal lesions by OCTA en face images is attributed to low or unbalanced blood flow within the polypoidal lesions, in addition to segmentation errors due to the presence of exudation, hemorrhage and PEDs^9,15^. The present study included some cases in which the blood flow signals within polypoidal lesions after the loading-phase treatment were tiny and difficult to assess employing en face images. Although OCTA en face images are intuitively understandable because they are similar to ICGA images, B-scan images might be more useful for detailed evaluation of blood flow signals within polypoidal lesions.

In 13.9% of cases in which ICGA showed complete regression of the polypoidal lesion after the loading-phase treatment, blood flow signals within polypoidal lesions were detected on OCTA B-scan images. In contrast, in all cases in which OCTA showed disappearance of the blood flow signals within polypoidal lesions, ICGA also showed complete regression of the polypoidal lesions. The PLEX Elite 9000 used in the present study can detect low blood flow because it acquires flow signals using an algorithm based on amplitude and phase. Moreover, the 3 × 3 mm OCTA used in this study detects blood flow signals from four OCT scans taken at the same site, thus enabling imaging with high blood flow sensitivity, while OCTA with other scan areas detects blood flow signals from two OCT scans. Therefore, OCTA with a 3 × 3 mm scan area on the PLEX Elite 9000 may be able to detect even low blood flow that cannot be detected with ICGA. Furthermore, the OCTA B-scan displays the detected blood flow signals in a uniform color, regardless of the flow rate, facilitating evaluation of the presence of blood flow, also thereby possibly contributing to the high blood flow sensitivity of OCTA. However, tiny blood flow signals must be carefully differentiated from projection artifacts.

This study has several limitations. First, this was a retrospective, single-center study with a relatively small number of patients all of whom were Japanese. Moreover, the study period was only one year. Furthermore, the 3 × 3 mm scan area employed did not include the entire BVN in some cases, such that the association between treatment outcomes and BVN blood flow was not evaluated. Additionally, OCTA was performed only at baseline and again at 3 months after the initial treatment, thereby limiting the evaluation of longitudinal changes in blood flow signals within polypoidal lesions.

In conclusion, PCV cases that showed disappearance of blood flow signals within polypoidal lesions on OCTA after the loading-phase treatment with IVBr showed favorable 1-year treatment outcomes. Evaluating blood flow signals within polypoidal lesions by OCTA may allow noninvasive prediction of the treatment outcomes of PCV.

## Methods

We obtained approval from the Institutional Review Board of Gunma University Hospital and adhered to the guidelines of the Declaration of Helsinki in performing this study. Informed consent was obtained from all individual participants included in the study. We retrospectively studied PCV cases who started IVBr treatment during the period from June 2020 to May 2022 at Gunma University Hospital and completed a 1-year treatment protocol with IVBr.

Before starting the IVBr treatment, all patients underwent complete ophthalmological examinations, including fundus examination employing slit-lamp microscopy with a noncontact fundus lens (SuperField lens; Volk Optical, Inc., Mentor, OH, USA), color fundus photography (Canon CX-1; Canon, Tokyo, Japan), SS-OCT (DRI OCT-1 Atlantis and Triton; Topcon Corp, Tokyo, Japan), fluorescein angiography (FA), ICGA, spectral-domain (SD) OCT (Spectralis HRA + OCT; Heidelberg Engineering, Heidelberg, Germany), and OCTA (PLEX Elite 9000; Carl Zeiss Meditec, Dublin, CA, USA). For the SS-OCT examination, we obtained B-mode images of horizontal and vertical line scans (12 mm) as well as 12 radial scans (9 mm) through the fovea. Moreover, horizontal B-mode images through the polypoidal lesions were obtained by SD-OCT with an angle of 30 degrees. FA, ICGA, SD-OCT, and OCTA were performed before and 3 months after the initial treatment, and other examinations were performed at each visit. Whether polypoidal lesions were present was evaluated on ICGA and B-mode OCT images, i.e., polyp-like choroidal vessel dilation on ICGA and sharply-peaked PED on B-mode OCT. We performed OCTA volume scanning, i.e., 300 × 300 pixels in a 3 × 3 mm area. The presence or absence of blood flow signals within polypoidal lesions detected by ICGA was evaluated with horizontal and vertical sectioned OCTA B-scan slices taken every 20 µm. We excluded cases in which the entire polypoidal lesion was not included in the scanning area and those in which it was difficult to evaluate the blood flow signal within the polypoidal lesion due to massive subretinal hemorrhage or hemorrhagic PED.

All cases received 3 monthly IVBr (6 mg/0.05 ml) treatments in the loading phase. In the maintenance phase, all cases received a TAE regimen with IVBr. The injection interval was extended by 4 weeks if there were no exudative changes and shortened by 4 weeks if any exudative changes were detected. The injection interval in the maintenance phase was a minimum of 8 weeks and a maximum of 16 weeks. BCVA, FT, and CCT were determined at each visit. Polypoidal lesion regression was assessed 3 months after the initial treatment, i.e., one month after the loading-phase treatment, utilizing ICGA. On ICGA, if no polypoidal lesions were detected, the eye was categorized as showing regression; otherwise, as having residual lesions. BCVA was determined with manifest refraction and recorded as decimal values and then converted to the logarithm of the minimal angle of resolution (logMAR) units. FT, CCT, and the height of the polypoidal lesion were measured on B-mode images employing the caliper measurement tool that is installed in the OCT system. FT was defined as the distance between the internal limiting membrane and the RPE surface at the fovea. CCT was defined as the distance between Bruch’s membrane and the margin of the choroid and sclera under the fovea. The height of polypoidal lesion was defined as the maximum perpendicular distance between Bruch’s membrane and the RPE surface at the polypoidal lesion showing maximum protrusion. We compared treatment outcomes between the groups with persistence versus disappearance of blood flow signals within the polypoidal lesions 3 months after the initial treatment. Persistence was defined as blood flow signals within polypoidal lesions being detected in two or more slices of the OCTA B-scan regardless of the signal size, and disappearance as detectable blood flow signals in just one slice or none at all. In cases with multiple polypoidal lesions in a single eye, we classified the eye into the signal persistence group whenever such a signal was detected in even one of them. If it was difficult to identify polypoidal lesions after the loading-phase treatment employing OCTA B-scans due to changes in the shapes of PED, we identified the area where polypoidal lesions were present before treatment by using a merkmal of retinal vessels on en face OCTA and then evaluated blood flow signals within the PED in that area. The blood flow signals were independently evaluated by two experienced retinal specialists (J. Hoshino and H. Matsumoto). If their evaluations of blood flow signals differed, the decision was reached by consensus between these two specialists.

For statistical analyses, the χ2 independence test was applied to determine differences in gender between the two groups. The Wilcoxon signed-rank test was used for intergroup comparisons of BCVA, FT, and CCT. We used the Mann–Whitney U test to compare differences in BCVA, FT, CCT, the height of the polypoidal lesion, number of injections, and the injection interval between the two groups. We employed Excel (Microsoft, Redmond, WA, USA) with the add-in software Statcel4 for the data analyses^30^. A value of *P* < 0.05 was taken to indicate a statistically significant difference. Age, BCVA, FT, CCT, the height of the polypoidal lesion, number of injections, and injection interval are presented as averages ± standard deviation (median (min, max)).

## Data Availability

The datasets used and/or analyzed during the current study are available from the corresponding author upon reasonable request.
